# Giving parents support: a randomized trial of peer support for parents after NICU discharge

**DOI:** 10.1038/s41372-022-01341-5

**Published:** 2022-03-08

**Authors:** Karen Fratantoni, Lamia Soghier, Katherine Kritikos, Juliana Jacangelo, Nicole Herrera, Lisa Tuchman, Penny Glass, Randi Streisand, Marni Jacobs

**Affiliations:** 1 Division of General and Community Pediatrics, Children’s National Hospital, Washington, DC USA; 2 Center for Translational Science, Children’s Research Institute, Children’s National Hospital, Washington, DC USA; 3 Department of Neonatology, Children’s National Hospital, Washington, DC USA; 4 Division of Biostatistics and Study Methodology, Children’s Research Institute, Children’s National Hospital, Washington, DC USA; 5 Department of Adolescent and Young Adult Medicine, Children’s National Hospital, Washington, DC USA; 6 Department of Psychology and Behavioral Health, Children’s National Hospital, Washington, DC USA

**Keywords:** Paediatrics, Outcomes research

## Abstract

**Background:**

Peer support during inpatient hospitalization has been recommended for NICU parents and can improve maternal mental health. Less is known about the impact of peer support after NICU discharge on parental mental health and infant healthcare utilization.

**Methods:**

Three hundred families of infants approaching discharge from a Level IV NICU were randomized to receive a care notebook (control) or care notebook plus peer support for 12 months (intervention). Participants reported on measures of stress, depression, anxiety, self-efficacy, and infant healthcare utilization. Analysis compared outcomes between control and treatment groups.

**Results:**

Parental depression, anxiety, stress, and self-efficacy improved significantly for all participants, yet there were no differences between control and intervention groups. Infant ED visits, hospitalizations, immunization status, and developmental status at 12 months did not differ between groups.

**Conclusions:**

Peer support after NICU discharge did not improve self-reported parental mental health measures or infant healthcare utilization.

**Clinical trial registration:**

NCT02643472.

## Introduction

Peer support has been shown to reduce parental stress, anxiety and depression for parents of preterm infants in the NICU [1, 2] and for parents of children with chronic conditions [3]. While recommended as part of NICU family centered care [4, 5], evidence about the impact of post NICU discharge support on parental mental health is lacking.

An infant’s NICU stay is stressful and anxiety provoking for parents, and many feel ill-equipped to handle the experience [6–8]. Maternal depression and anxiety can be associated with infant feeding problems and can impact parenting decisions and practices [9]. Children of mothers with depression may not receive timely age-appropriate well child visits and immunizations and are more likely to receive Emergency Department (ED) care [10]. Mothers who are depressed may have lower maternal self-efficacy, which is associated with increased infant hospitalization [11].

For as many as 30% of NICU parents, mental health concerns persist well into the first year following their infant’s birth [12, 13]. After discharge, many parents feel socially isolated and lack optimal support [14]. While improving health outcomes for premature and medically complex infants has historically been the primary focus [7, 15, 16], NICUs should also pay attention to the psychosocial needs of parents during hospitalization [17–19] and after discharge [20]. Peer support from a parent with lived experience [21] may be helpful in mitigating some of the stressors NICU parents face after discharge.

In the peer support model, the caregiver shares a similar lived experience with the care recipient, and the care provided usually involves sharing of information/resources, emotional support, and encouragement [22]. Peer support is flexible in its approach. Services can be provided in person, by phone or email, in groups or individually and in different settings.

We implemented a large randomized controlled trial of post NICU discharge peer support to examine the impact of peer support on parental and infant outcomes [23, 24]. This intervention was based upon the primary care parent navigator program at Children’s National Hospital, which employs parents of children with special healthcare needs (CSHCN) to provide peer support to parents of CSHCN who are seen in the primary care center. The main aim of the Giving Parents Support study was to determine if peer support could improve parental mental health, including self-efficacy, stress, anxiety, and depression among NICU parents during the 12 months after discharge. The secondary aim was to determine if peer support could impact infant health outcomes during the 12 months after NICU discharge.

## Methods

### Design, setting, and participants

This study is a randomized controlled trial of structured parental peer support after NICU discharge. All infants approaching discharge from the Children’s National NICU between January 4, 2016 and February 24, 2017 were assessed for eligibility. Children’s National Hospital NICU is a large, level IV [25] NICU at a freestanding children’s hospital without a co-located delivery room; infants are admitted directly from the ED or transferred from other institutions for surgical management or subspecialty care.

During the period of identification and enrollment, the study staff reviewed the NICU census daily and completed chart review for each new admission. A parent was eligible for enrollment if they were English speaking, ≥18 years of age, planned to remain in the Washington, DC metropolitan area for the 12-month study period, and had an infant with an anticipated discharge within 2 weeks. Initial enrollment criterion requiring infants to have a NICU length of stay (LOS) ≥14 days as a proxy for medical complexity was liberalized early in the study to include infants with any LOS, as all infants admitted to this referral NICU are medically complex. One parent per infant was enrolled, and each family was asked to self-select the primary caregiver to participate. Eligible parents provided written, informed consent. This study was approved by the Children’s National Hospital Institutional Review Board and registered with Clinicaltrials.gov (NCT02643472). Details of the clinical trial protocol have been published elsewhere [23].

### Randomization

Following enrollment, participants were randomized to either the intervention or control group (1:1). Randomization occurred after completion of the baseline surveys, stratified by infant birth weight (BW) (≥1500 grams or <1500 grams). Those in the ≥1500 g stratum were randomized in permuted blocks of 2 or 4 with random variation of the blocking number. As fewer infants were expected in the <1500 g stratum, random permuted blocks of block size 2 were used. The randomization schedule was created in Stata/SE 13.1 (StatCorp LP, College Station, TX) and implemented in the Research Electronic Data Capture (REDCap) ^TM^ online system [26]. After enrollment and administration of the baseline surveys, the participant randomization determination was displayed in REDCap ^TM^ to the research assistant, who immediately shared it with the participant. Efforts were made to blind principal and co-investigators throughout the study.

Parents randomized to the intervention group were assigned a peer navigator and received a care notebook prior to NICU discharge. Parents randomized to the control group received a care notebook before NICU discharge only. The care notebook included information about community resources and had sections to record information about the infant’s care (e.g., medications, appointments, care plans, seizure logs, therapy goals, dietary schedules, etc.) to share among caregivers or with medical/therapeutic providers.

### Intervention

In planning the intervention, focus groups were used to determine needs of families after NICU discharge and findings informed the intervention and the training curriculum. Parents with previous NICU experience were hired as peer navigators to provide emotional support, access to community resources, assistance with navigating the health system, and parent empowerment. Peer navigators received specific training in communication skills, establishing and maintaining boundaries, and NICU specific medical terminology. They were trained to identify resources to meet parents’ needs and assist in preparing for, making, and keeping specialist and primary care appointments; answer questions about insurance coverage, medical equipment and related supplies; and to serve as a liaison between parents and physicians, therapists, pharmacists, and medical supply companies. Peer navigators were a small group of 3 who partnered with multiple families each. Peer navigators attempted initial contact with parents before discharge, and then within 2 weeks post-discharge and monthly thereafter for 12 months by phone, email, or in person at an in-hospital medical appointment. As some parents would require more support than others, the total effect of the intervention, rather than the individual use, was measured.

### Measures

Parental mental health outcomes were self-reported using standardized, validated measures. Surveys were verbally administered by study staff in person at baseline data collection, and by phone at follow-up time-points of 1 week, 1 month, 3 months, 6 months, and 12 months post-discharge and entered directly into a REDCap^TM^ database. If participants were not able to complete the follow-up surveys by phone, they were given the opportunity to complete surveys online using the survey function in REDCap^TM^.

The Perceived Stress Scale (PSS-10) assessed general stress over the last month [27–32]. Specific stress related to parenthood was measured through the Parental Stress Scale (PSS) [33, 34]. The Perceived Maternal Parenting Self-Efficacy Scale (PMPS-E) measured parental perception of self-efficacy [35–38]. Parental depressive symptoms were assessed using the 10-item Center for Epidemiologic Studies Depression Scale (CES-D 10), a shortened version of the 20-item scale, the Center for Epidemiologic Studies Depression Scale (CES-D 20) [31, 39–43]. The Study PI called parents with elevated depression scores (CESD-10 ≥ 10) within 1 week of survey completion and mailed a letter with local mental health resources. Anxiety was measured through the state portion of the State-Trait Anxiety Inventory (STAI Y-1) [2, 32, 44–54].

Infant outcomes included ED visits, hospitalizations, and immunization status. At each visit, parents were asked to self-report the number of ED and hospitalization occurrences, and total number of occurrences over the 12-month study periods was summed separately. Infants were further classified as having none or any ED visits or hospitalizations to account for small numbers of repeats visits. Immunization status was assessed at 12 months of age and classified by receipt of the following three vaccines: (1) diphtheria, tetanus, and pertussis (DTaP), (2) pneumococcal 13-valent conjugate (PVC13), and (3) Haemophilus influenzae type b (Hib). Infants receiving three DTaP and three PVC13 vaccines were considered fully immunized. Infants receiving two doses of the monovalent Hib vaccine or three doses of the combination Hib vaccine were considered fully immunized [55]. Immunization status was obtained from the infant’s primary care provider or state records (DC and Maryland).

Infant developmental progress at the 12-month visit was measured using the Bayley Scales of Infant and Toddler Development^®^, Third Edition (Bayley III^®^) [56]. During the evaluation, a licensed psychologist provided families with immediate feedback, suggestions for ways to promote the baby’s development at home and relevant referrals. A formal letter describing results of the assessment was sent to the family and, with parental permission, to the primary care pediatrician.

### Analysis

Analyses were specified as intent-to-treat analyses to assess the effects of the peer mentor intervention on psychosocial outcomes. It was expected that randomization with stratification on the key confounder of BW (≥1500 g or <1500 g) would result in a balanced distribution of demographic and risk factors between groups. However, prior to analysis, assessment of imbalances between groups was conducted.

For all scales, mean imputation, rounded up to the next whole number, was used to get a more complete outcome score if ≤10% of the items were missing. Documentation for this method is provided by the authors of two of the outcome scales [57, 58] and was carried across to the other outcomes for consistency.

Differences in psychosocial measures over time between groups was estimated using generalized estimating equation (GEE) to account for correlation between repeated measures. GEE models are reasonably robust to missing data, as is expected to occur during longitudinal studies, and allow all data to be included, regardless of whether visits are missed. Several covariance structures were tested; the structure with the best fit according to the lowest quasi-information criterion (QIC) was selected. Intervention group-by-time cross-product terms were evaluated in all models enabling estimation of differences in trajectories between groups over time, as well as estimates of differences in scores by time-point. Differences between groups at 3 months was pre-specified as determination of short-term intervention effects, 6 months as intermediate effects, and differences between groups at 12 months allowed assessment of long-term effects. Difference between groups was considered statistically significant when the 95% CIs around the difference did not include 0. Actual time in months from NICU discharge to assessment was included in all GEE models to account for any variations in visit timing. Models were used to plot trajectories over time by group, with 95% confidence intervals (CIs) around each time-point estimate. All analyses were completed using SAS 9.4.

## Results

**Fig. 1:**
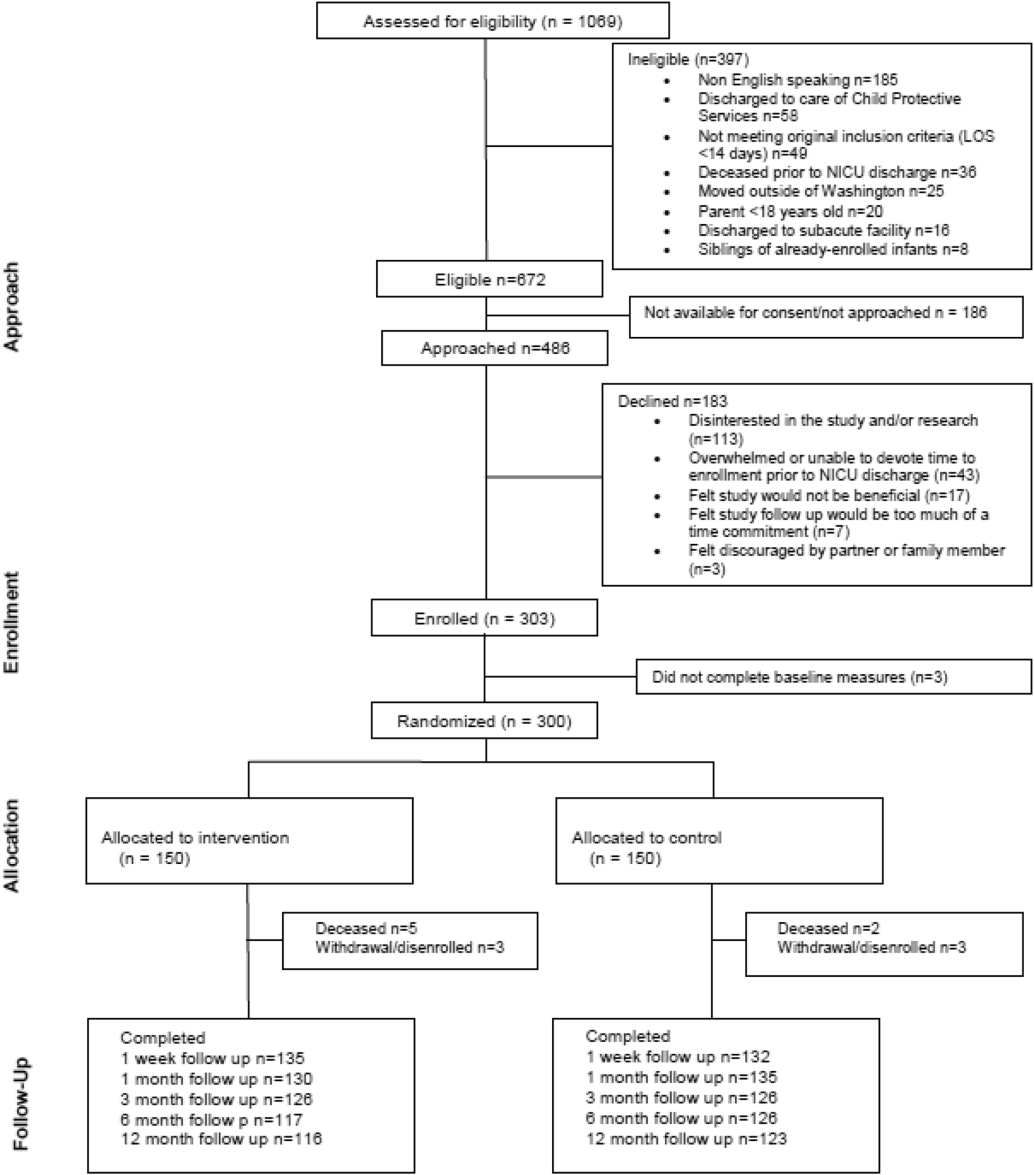
CONSORT Diagram. Diagram of participant eligibility, assessment, enrollment and randomization [24].

A total of 303 families enrolled in the study, and 300 were randomized (150 per group) (CONSORT diagram Fig. 1). Overall follow-up rate was 88%. Only 2% of participants were missing all follow-up data.

No differences in demographic or patient characteristics were noted between randomization groups (Table 1). Differences in outcome measures at baseline were minor, ranging from 0.1 points to 1.2. One participant completed the baseline assessments after discharge and was excluded from baseline scale analyses (*n* = 299).

**Table 1:** Infant and parent characteristics by group.

Demographics	Overall (*n* = 300)	Control (*n* = 150)	Intervention (*n* = 150)	*p* value^a^
	*n* (%)	*n* (%)	*n* (%)	
Infant characteristics				
Gestational age, weeks (mean, SD)	35.5 (4.8)	35.4 (4.8)	35.7 (4.8)	0.51
Birth weight, grams (mean, SD)	2540 (1047)	2521 (1041)	2559 (1056)	0.70
NICU length of stay, days (mean, SD)	34 (39)	34 (38)	35 (40)	0.94
Sex				0.82
Male	174 (58.0)	88 (58.7)	86 (57.3)	
Female	126 (42.0)	62 (41.3)	64 (42.7)	
Race				0.42
White	117 (39.0)	56 (37.3)	61 (40.7)	
Black	133 (44.3)	66 (44.0)	67 (44.7)	
Asian	17 (5.7)	11 (7.3)	6 (4.0)	
American Indian/Pacific Islander	8 (2.7)	6 (4.0)	2 (1.3)	
Mixed race/not reported	25 (8.3)	11 (7.3)	14 (9.3)	
Ethnicity				0.52
Hispanic	23 (7.7)	13 (8.7)	10 (6.7)	
Non-Hispanic	277 (92.3)	137 (91.3)	140 (93.3)	
Parent characteristics				
Age, years (mean, SD)	30.1 (6.5)	30.4 (6.5)	29.9 (6.5)	0.41
Gender				0.10
Male	33 (11.0)	21 (14.0)	12 (8.0)	
Female	267 (89.0)	129 (86.0)	138 (92.0)	
Education				0.48
High school or less	77 (25.7)	34 (22.7)	43 (28.7)	
Vocational/some college	87 (29.0)	46 (30.7)	41 (27.3)	
College or more	136 (45.3)	70 (46.6)	66 (44.0)	
Relationship status				0.43
Single	49 (16.3)	22 (14.7)	27 (18.0)	
Married/partnered	251 (83.7)	128 (85.3)	123 (82.0)	
Other children at home				0.59
None	129 (43.0)	68 (45.3)	61 (40.7)	
At least one other	171 (57.0)	128 (85.3)	89 (59.3)	
Baseline scale scores (mean, SD)				
PMP-SE	70.3 (8.1)	70.3 (7.9)	70.2 (8.4)	1.00
PSS	30.2 (7.9)	29.6 (8.0)	30.8 (7.9)	0.16
PSS-10	17.4 (7.0)	17.1 (7.2)	17.6 (6.8)	0.46
STAI	34.7 (12.3)	34.4 (12.2)	35.0 (12.4)	0.62
CES-D 10	9.6 (5.4)	9.4 (5.7)	9.7 (5.1)	0.60

*PMPS-E* Perceived Maternal Parenting Self-Efficacy Scale, *PSS-10* Perceived Stress Scale, *PSS* Parental Stress Scale, *STAI Y-1* State-Trait Anxiety Inventory (State), *CES-D 10* 10-item Center for Epidemiologic Studies Depression Scale.

^a^*p* values based on Chi-square for categorical measures and *t* test for continuous measures.

### Parental mental health

**Fig. 2:**
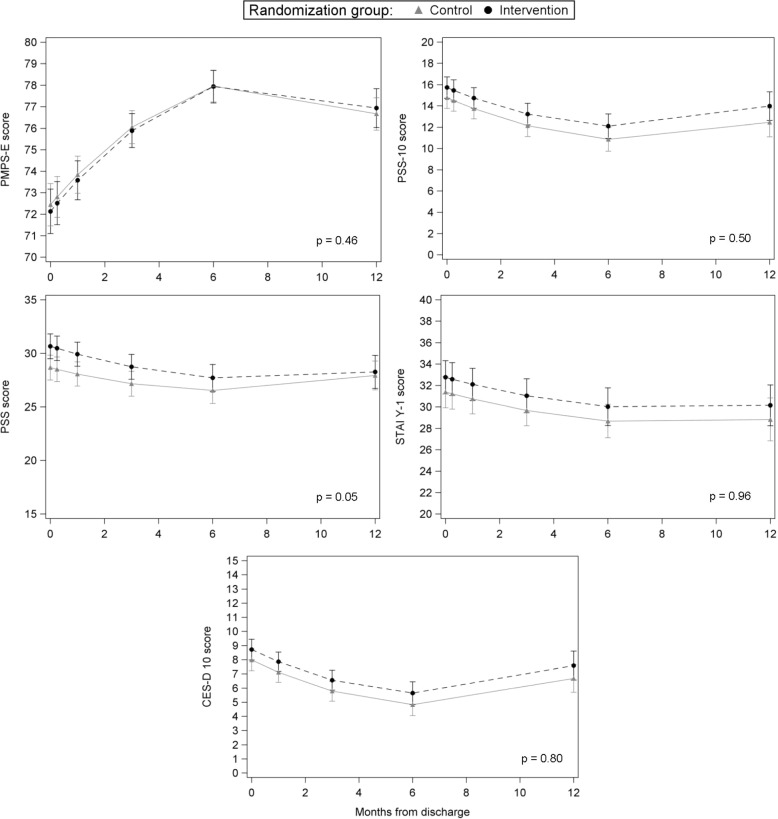
Trajectory of Mental Health Outcomes Between Groups. Parental self-efficacy, perceived stress, parental stress, anxiety and depression among those in intervention and control over the 12 months after NICU discharge.

For both intervention and control groups, mental health outcomes improved over time (*p* < 0.0001), with a slight decline or leveling off noted during the end of follow-up (Fig. 2). Overall, no differences in mental health trajectories over the 12-month-study period were seen between groups. Slight trajectory differences were noted for parental stress (PSS, *p* = 0.05); however, this appears driven by the slight increase in scores from 6 to 12 months in the control group to match scores in the intervention group. At all time-points of interest (3, 6, and 12 months), stress, anxiety, and depression scores were slightly lower in the control group but not clinically significant (see Table 2). Parental stress was statistically significantly lower in the control group at 3 months only (mean difference = −1.58 points, 95% CI −3.15, −0.01).

**Table 2:** Differences in scale scores between control and intervention groups by time-point.

Psychosocial measure	Difference between control and intervention groups^a^					
	Short-term effects (3 months)		Medium term effects (6 months)		Long-term effects (12 months)	
	Mean	(95% CI)	Mean	(95% CI)	Mean	(95% CI)
PMPS-E	0.16	(−0.95, 1.28)	0.02	(−0.96, 1.00)	−0.27	(−1.40, 0.86)
PSS-10	−1.09	(−2.43, 0.24)	−1.24	(−2.65, 0.17)	−1.54	(−3.40, 0.32)
PSS	−1.58	(−3.15, −0.01)	−1.16	(−2.80, 0.47)	−0.33	(−2.35, 1.68)
STAI Y-1	−1.36	(−3.35, 0.63)	−1.35	(−3.42, 0.73)	−1.32	(−3.99, 1.36)
CES-D 10	−0.78	(−1.74, 0.18)	−0.82	(−1.84, 0.20)	−0.90	(−2.29, 0.48)

*PMPS-E* Perceived Maternal Parenting Self-Efficacy Scale, *PSS-10* Perceived Stress Scale, *PSS* Parental Stress Scale, *STAI Y-1* State-Trait Anxiety Inventory (State), *CES-D 10* 10-item Center for Epidemiologic Studies Depression Scale.

^a^Based on least squares means estimates from GEE models controlling for time from NICU discharge at each assessment and non-linear time effects.

### Infant health outcomes

Full immunization was relatively high overall (73.5%), and no difference in immunization status was observed between groups (Table 3); approximately one-quarter of both groups (25.2% of controls and 27.9% of the intervention group) were not fully immunized. Twenty-three percent of infants were hospitalized over the first year, and of those hospitalized, 61% were hospitalized once and 24% twice. Proportion hospitalized did not differ between groups (22% vs. 24%, *p* = 0.63). While ED visits were relatively common during the first year (44% visited the ED at least once), more than half (51%) of those who visited the ED went only once while an additional 28% went twice. ED utilization did not differ between groups (42% vs. 47%, *p* = 0.35). No difference in the number of hospitalizations or ED visits was noted between study groups (median = 0 in both groups, *p* = 0.64 and *p* = 0.43 respectively, data not shown). No differences in infant development at 1 year were seen.

**Table 3:** Healthcare utilization and infant development at 12 months.

Healthcare utilization metric	Overall^a^	Control	Intervention	*p* value^b^
	*n* (%)	*n* (%)	*n* (%)	
Fully immunized				0.60
No	74 (26.5)	36 (25.2)	38 (27.9)	
Yes	205 (73.5)	107 (74.8)	98 (72.1)	
Hospitalized				0.63
No	225 (77.1)	115 (78.2)	110 (75.9)	
Yes	67 (22.9)	32 (21.8)	35 (24.1)	
ED visit				0.35
No	163 (55.8)	86 (58.5)	77 (53.1)	
Yes	129 (44.2)	61 (41.5)	68 (46.9)	
**Infant development**^c^	**Mean (SD)**	**Mean (SD)**	**Mean (SD)**	***p*** **value**^**d**^
Cognitive	97.98 (16.97)	97.83 (16.25)	98.13 (17.13)	0.92
Language	87.71 (16.37)	88.49 (15.33)	86.89 (17.49)	0.58
Motor	91.52 (17.44)	91.26 (16.02)	91.79 (18.98)	0.87

^a^Eight missing immunization information, 13 excluded (7 deaths, 5 withdrawals, 1 disenrolled).

^b^*p* value based on Chi-square.

^c^Bayley III^®^: Bayley Scales of Infant and Toddler Development^™^ Domain.

^d^*p* value based on *t* test.

Given the lengthy study period, sensitivity analyses were conducted to evaluate the potential impact of loss to follow-up (LTFU). Parents who could not be reached at 12 months tended to be younger, lower educated, single, and had higher self-efficacy at baseline, though no differences in infant characteristics were observed.

## Discussion

Peer support interventions have been utilized in the NICU to improve parent competence [59] and maternal mental health [1, 2]. Our peer navigators offered peer support and empathetic listening, scheduled appointments and attended visits, shared resources, and encouraged families to recognize their abilities and successes in caring for their child. While parental mental health improved during the first year after discharge, those who received peer support after discharge showed no differences in self-efficacy, general stress, anxiety, or depression compared with parents who did not. Self-efficacy was high at baseline and increased significantly over the study period in all groups.

Several reasons for these trial results exist. The timing of introduction between parents and peer navigators may have impacted our findings. Preliminary focus groups identified the need to introduce peer support during discharge, rather than earlier in the stay, to prevent interference in clinical care. However, consent and enrollment close to discharge was difficult to operationalize, and peer support was often introduced immediately before discharge, a busy time for parents as they prepare for transition, finish necessary training, and actively assume the primary caregiving role. It is possible that earlier introductions would have provided more time for relationship and trust building.

All eligible parents, instead of those with specific pre-identified needs, were enrolled. Peer navigators made scheduled contact with parents; however, the amount and type of support provided varied. This may have decreased the efficacy of the intervention. Affleck et al. showed that post NICU discharge support had a positive effect on mothers’ sense of competence and interaction with their infants if they acknowledged the need for support but had a negative effect on those who needed less support [60]. Matching peer support to parents based on need, child’s medical condition, demographics, culture or language has been associated with improved maternal mental health outcomes. Preyde and Ardal showed that peer support for parents of premature infants based on infant medical condition, language/ethnicity and geographic location showed a decrease in maternal stress, anxiety and depression and an increase in social support [2]. Peer navigators in our intervention had general NICU experience but were not matched with participants by diagnosis or demographics, which may have decreased the impact of the intervention.

Participants in the control group received a care notebook, regular communication with our research team for interval data collection, and resources for elevated depression scores. It is possible that these interactions were enough to diminish the isolation, stress and depression of newly discharged parents; hence, the peer support intervention may not have been additionally impactful. A high level of baseline self-efficacy in both groups, due to support systems in place in the NICU and optimal pre-discharge preparation, may have also made it difficult to detect a rise in self-efficacy in either group.

There were no differences in infant outcomes, specifically hospitalizations, ED visits, immunization status, and developmental progress, between groups at 12 months. Previous studies have shown maternal depressive symptoms can impact child hospitalizations [61–65], ED visits [64, 66], and decrease in up-to-date immunization [10]. Participants in our study had few ED visits and hospitalizations overall which likely explains the lack of findings between the groups. Maternal depression can impact optimal language development in toddlers [66]. As there were no differences in depressive symptoms between those in our study who did or did not receive peer support, it is not surprising that there were similarly no differences in infant outcomes.

Strengths of the study include a randomized controlled trial design, large sample size, mixture of term and preterm infants, diversity of the participant population, a long follow-up time and high retention rate. Limitations included the lack of complete standardization of the intervention, as amount and type of support varied among participants, and the inability to compare individual participant outcomes with amount or quality of peer support.

NICU families should receive peer support [4] as a component of family centered care [5, 67]. This study does not contradict this recommendation but shows that general peer support after discharge does not specifically improve measures of parental mental health or significantly alter patterns of infant healthcare utilization.

## Conclusions

Giving Parents Support investigated the effects of peer support after NICU discharge on parental mental health and infant healthcare utilization. Stakeholder engagement informed the intervention, which began at NICU discharge and continued over the course of 12 months. Although parental mental health improved overall, no differences were detected between groups. Future studies should investigate the impact of the timing of introduction of peer support, identifying those parents who would benefit most, and matching parents with navigators based on meaningful characteristics.
