# Correction to: Giving parents support: a randomized trial of peer support for parents after NICU discharge

**DOI:** 10.1038/s41372-022-01402-9

**Published:** 2022-04-21

**Authors:** Karen Fratantoni, Lamia Soghier, Katherine Kritikos, Juliana Jacangelo, Nicole Herrera, Lisa Tuchman, Penny Glass, Randi Streisand, Marni Jacobs

**Affiliations:** 1 Division of General and Community Pediatrics, Children’s National Hospital, Washington, DC USA; 2 Center for Translational Science, Children’s Research Institute, Children’s National Hospital, Washington, DC USA; 3 Department of Neonatology, Children’s National Hospital, Washington, DC USA; 4 Division of Biostatistics and Study Methodology, Children’s Research Institute, Children’s National Hospital, Washington, DC USA; 5 Department of Adolescent and Young Adult Medicine, Children’s National Hospital, Washington, DC USA; 6 Department of Psychology and Behavioral Health, Children’s National Hospital, Washington, DC USA

**Keywords:** Paediatrics, Outcomes research

Correction to: *Journal of Perinatology* 10.1038/s41372-022-01341-5, published online 08 March 2022

Due to an error during the implementation of the corrections, the reference [24] was deleted in the legend of Figure 1. The original article has been corrected.

